# Can income-based co-payment rates improve disparity? The case of the choice between brand-name and generic drugs

**DOI:** 10.1186/s12913-019-4598-8

**Published:** 2019-11-01

**Authors:** Yuki Ito, Konan Hara, Byung-Kwang Yoo, Jun Tomio, Yasuki Kobayashi

**Affiliations:** 1 Department of Public Health, Graduate School of Medicine, The University of Tokyo, 7-3-1 Hongo, Bunkyo-ku, Tokyo, 113-0033 Japan; 2 Department of Public Health Sciences, University of California, Davis, School of Medicine, One Shields Ave., Medical Sciences 1C, Davis, CA 95616 USA

**Keywords:** Co-payment rate, Generic drugs, Disparity, Pharmaceuticals

## Abstract

**Background:**

Higher income population tend to prefer brand-name to generic drugs, which may cause disparity in access to brand-name drugs among income groups. A potential policy that can resolve such disparity is imposing a greater co-payment rate on high-income enrollees. However, the effects of such policy are unknown. We examined how patients’ choice between brand-name and generic drugs are affected by the unique income-based co-payment rates in Japan; 10% for general enrollees and 30% for those with high income among the elderly aged 75 and over.

**Methods:**

We drew on cross-sectional price variation among commonly prescribed 311 drugs using health insurance claims data from a large prefecture in Japan between October 2013 and September 2014 to identify between-income-group differences in responses to differentiated payments.

**Results:**

Running 311 multivariate logistic regression models controlling individual demographics, the median estimate indicated that high-income group was 3% (odds ratio = 0.97) less likely to choose a generic drug than the general-income group and the interquartile estimates ranged 0.92–1.02. The multivariate feasible generalized least squares model indicated high-income group’s higher likelihood to choose brand-name drugs than the general-income group without co-payment rate differentiation (*p* < 0.001). Such gap in the likelihood was attenuated by 0.4% (*p* = 0.027) with an US$1 increase in the difference in additional payment/month for brand-name drugs between income groups — no gap with US$10 additional payment/month. This attenuation was observed in drugs for chronic diseases only, not for acute diseases.

**Conclusions:**

Income-based co-payment rates appeared to reduce disparity in access to brand-name drugs across income groups, in addition to reducing total medical expenditure among high-income group who shifted from brand-name drugs to generic ones due to larger drug price differences.

## Background

Increasing health expenditures is a serious concern among developed countries. During 2009–2016, Organisation for Economic Co-operation and Development (OECD) countries experienced a 1.4% annual average growth rate in per capita health expenditure [1], highlighting the growing need for policies which can minimize the health expenditure growth rate without harming clinical outcomes. At the same time, disparity in access to healthcare has become a major issue in reforming healthcare systems among OECD countries [2]. This indicates that health-expenditure-based policies should not worsen disparities among the subgroups of the population to sustain feasibility of the policies.

To take control of health expenditure without worsening disparity in access to healthcare, among other objectives, all 36 OECD countries have some form of publicly subsidized health insurance that covers some or all of the population [1]. Although most of OECD countries have imposed a progressive income-based premium and/or a tax to finance a public insurance system, high-income enrollees are reported to have better access to healthcare than low-income ones given the common co-payment rates if their health conditions are equal [3]. This general observation is consistent with Becker’s (2007) assumption that even after paying a premium, the willingness to pay for healthcare services, i.e., access to healthcare for the same health conditions, differs by income [4]. Our overall research question is whether income-based co-payment rates can reduce the disparity in access to healthcare services across income groups. Specific hypotheses are explained hereafter.

To consider the impact of income-based co-payment rates, we focused on the choice between brand-name and generic drugs. When drugs are no longer under the legal obligations of a patent, the generic versions of these drugs are sold at lower prices. Although brand-name and generic drugs are assumed to have the same level of efficacy [5], higher income population may tend to prefer brand-name to generic drugs even if co-payment rate is equal across income groups [6], which would cause disparity in access to brand-name drugs among income groups (Hypothesis 1). Therefore, imposing a greater co-payment rate on high-income enrollees can reduce disparity in terms of the equal access to brand-name drugs (Hypothesis 2), in addition to reducing expenditure without harming health conditions. Our hypothesis 3 is that the impact of income-based co-payment may differ between drugs treating acute and chronic conditions.

As an example of income-based co-payment rates, we used Japan’s public universal insurance system where individuals aged 75 years or above are offered a 10% co-payment rate for enrollees with general income, whereas a 30% rate for enrollees with high income above a certain threshold. Since we know of no other study addressing the impact of income-based co-payment on the gap in the use of generic drugs between income groups, our empirical findings will help future insurance scheme reform in co-payment settings.

## Methods

### Study population

We used health insurance claims data provided by the Medical Care System of Latter-stage Elderly, which is the Japanese public insurance system for all the individuals aged 75 years or older. The data was comprised of enrollees living in one prefecture that includes one of Japan’s major metropolitan areas. The data was based on 1.3 million individual members as of March 2014 and covered the period between October 1, 2013, and September 30, 2014. The study population was all the enrollees included in this data who used outpatient care, and whose prescriptions were dispensed by an external pharmacy that can minimize the potential physicians’ influence on the choice of the drug [7, 8]. Furthermore, we excluded individuals whose responses to income-based co-payment rates could be seriously influenced by the receipt of free medical care financed by two types of public assistance programs for those (a) reached the maximum out-of-pocket spending cap (i.e., about 8000–12,000 Japanese Yen (JPY)/month) or (b) enrolled in a welfare program. The latter group of welfare recipients accounts for 1.7% of Japan’s population, which is considerably smaller than those of OECD countries [9, 10]. We exclude such individuals from our study because the marginal prices for brand-name and generic drugs are both zero for these individuals.

In Japan, dispensing generic drugs and generic substitution is governed by the following rules: Rules for Health Insurance-covered Medical Institutions and Physicians and Rules for Health Insurance-covered Dispensing Pharmacies and Pharmacists. There were some incentives for prescribing generic drugs for the medical institutions. During our study period, additional reimbursement was provided to the medical institutions when they wrote a prescription that included a drug prescribed in its name of the ingredient. The additional amount of reimbursement was 20 JPY per prescription. Also, there were some incentives for dispensing and suggesting generic drugs for the pharmacies. During our observation period, pharmacies could receive additional reimbursement for each prescription when their generic drug dispense rate within pharmacy-month was higher than a specified threshold. The additional amount of reimbursement and corresponding thresholds were: 50 JPY (rate > 22%), 150 JPY (rate > 30%), 190 JPY (rate > 35%). Also, to get a reimbursement for managing patients’ prescription history (410 JPY), there was a requirement to suggest the use of generic drugs to the patient in addition to other requirements such as checking patients’ compliance to their drugs’ instructions and drug information provision using a notebook for prescription record. Furthermore, marketing and promotion for prescription drugs were governed by Pharmaceutical Affairs Act during our study period. We note that this law was replaced by Pharmaceuticals and Medical Devices Act in November 2014. Main point of these laws is that it is prohibited to advertise and promote prescription drugs directly to consumers. Advertising and promotion for prescription drugs are limited to health care professionals.

In 2013, the proportion of generic drugs dispensed where both brand-name and generic drugs were available was 43% [11]. Common reasons why the patients choose the brand-name drugs at the pharmacy are: patients wanted to follow the prescriptions where the physicians wrote down the brand-name product name and patients were suspicious about the effectiveness of generic drugs [12]. In addition, 46% of the drugs were prescribed by the generic name, while 49% were prescribed by the brand-name [12].

### Analytical model 1: association between drug choices and co-payment rates

To analyze the association between the patients’ choice between brand-name/generic drug and the patients’ co-payment rate, we conducted separate logistic regressions for each of 311 drugs as follows:

1 $$ \boldsymbol{Logit}\left(\boldsymbol{P}\left({\boldsymbol{y}}_{\boldsymbol{ikt}}=\mathbf{1}\right)\right)={\boldsymbol{\mu}}_{\boldsymbol{k}}+{\boldsymbol{D}}_{\boldsymbol{it}}{\boldsymbol{\beta}}_{\boldsymbol{k}}+{\boldsymbol{X}}_{\boldsymbol{ikt}}{\boldsymbol{\gamma}}_{\boldsymbol{k}}, $$

where dependent variable *y*_*ikt*_ is a dummy variable indicating a generic version of drug *k* was dispensed for patient *i* at prescription timing *t*.

The key covariate *D*_*it*_ is a dummy variable indicating the patient *i* ’s co-payment rate is 30% (the reference co-payment is 10%) at timing *t*. The coefficient *β*_*k*_ is our primary interest. The co-payment rate becomes 30% if at least one elderly household member who is entitled to the Medical Care System of Latter-stage Elderly has a taxable income that exceeds 1.45 million JPY (100 JPY is approximately 1 US dollar (USD)). If the total income within the household (total of net incomes before basic deduction of all family members aged 70 or over) is below certain values, which depends on the composition of the household, enrollees can apply for reducing the rate to 10%. The median household income for enrollees with 30 and 10% co-payment rates are approximately 7 million and 1 million JPY. Among the study population, 0.19 million (14%) enrollees incurred 30% co-payment, which was higher than the national average (6%) [13].

We included a set of covariates for *X*_*ikt*_ such as age, sex, amount of drug prescribed at *t* (which is the total number of tablets/capsules of drug *k* per prescription), area of patient’s residence, total medical expenditure in a month (including prescription timing *t* but excluding spending on drugs), and total spending on drugs besides drug *k* in a month including prescription timing *t. μ*_*k*_ is a constant term. Since individuals tend to receive a prescription for the same drug repeatedly, we employed a generalized estimating equation (GEE) approach to account for the clustering responses among the same individuals. To model the within-individual correlation, we chose a working correlation matrix to have an exchangeable structure.

For the above analysis, 311 drugs were chosen according to the following criteria: (i) drugs in the form of tablets or capsules, (ii) drugs whose generic version existed in our observation period, (iii) drugs whose generic share based on the prescribed amount was at least 5%, (iv) drugs whose number of prescriptions exceeded 12,000, and (v) drugs which are for daily use. We treated drugs with the same ingredient but different forms (e.g., tablets and capsules) or unit doses (e.g., amlodipine 2.5 mg or amlodipine 5 mg) as distinct drugs.

### Analytical model 2: association between price difference and disparity in access to brand-name drugs

Next, we analyzed whether price difference (between brand-name and generic drugs) is associated with the disparity in access to brand-name drugs. We conducted ordinary least squares (OLS) regression of the estimated coefficient ${\hat{\beta}}_k$ from Eq. (1) on price difference, since ${\hat{\beta}}_k$ represents disparity. The unit of analysis was each of 311 selected drugs. The equation for OLS regression is:

2 $$ \hat{\beta_k}={\uptheta}_k+\delta {p}_k+{\varepsilon}_k, $$

where *p*_***k***_ is the price difference between brand-name and generic drug of drug *k*, *θ*_***k***_ is a constant term, and ***ε***_***k***_ is an error term. We reported all standard errors for OLS regression in heteroscedasticity-robust standard errors.

We defined price difference as the product of price difference per unit dose and average daily dose. The median and interquartile of price difference were 31 JPY and 16–58 JPY, respectively. In addition, we conducted feasible generalized least squares (FGLS) estimation that yields consistent standard error estimates, wherein the dependent variable is based on the estimates. The procedure follows previous literature [14, 15]. We detailed the FGLS estimation procedure in the Additional file 1. Furthermore, we divided the drugs into two groups on the basis of their usage: acute and chronic drugs, as defined in the literature [16]. A total of 59 drugs were classified as acute drugs. We separately conducted OLS and FGLS regressions of Eq. (2) for acute and chronic drugs. Furthermore, for robustness check, we conducted OLS and FGLS regressions of Eq. (2) for chronic drugs, adding further detailed drug category dummies as explanatory variables. These dummies represent four categories of the chronic drugs: drugs for alimentary tract and metabolism, cardiovascular system, nervous system, and others. The first three are the top three categories in terms of the number of chronic drugs included in our analysis.

### Interpretation of the parameters

We constructed an econometric model that predicts each patient’s choice between brand-name and generic drug, which is detailed in the Additional file 1. Under our econometric model, coefficient *β*_*k*_ of high-income group dummy from Eq. (1) addresses disparity in access to brand-name drugs (a coefficient of zero means no disparity). Negative *θ*_*k*_ from Eq. (2) suggests positive income elasticity of brand-name drug demand. On the other hand, positive *δ* from Eq. (2) suggests that disparity has improved under the Japanese income-based co-payment policy if income elasticity is positive. Intuitively, since larger brand-generic price difference implies larger out-of-pocket payment difference between income groups, positive *δ* means that a state of no disparity (*β*_*k*_ = 0) may be achieved under a certain out-of-pocket payment difference.

## Results

### Summary statistics

In our analysis of 311 drugs, we observed 1,075,819 distinct enrollees with 35,221,733 drug prescriptions. At the individual level, the median age was 81 years, while the interquartile range was 78–86 years. Females comprised 62% of our sample and 13% reported a co-payment rate of 30%. The median total health spending for outpatient care (sum of the amount spent by the enrollee and the insurer) per year, excluding expenditure on drugs, was 116,350 JPY and the total median spending on drugs per year was 121,838 JPY.

Table 1 presents the summary statistics for the top-five prescribed drugs in terms of the number of prescriptions: 100 mg tablet of rebamipide, 5 mg tablet of amlodipine, 15 mg oral disintegrating tablet of lansoprazole, 12 mg tablet of sennoside, and 0.5 mg tablet of etizolam. We saw a higher mean age, amount per prescription, and medical spending in the high-income group. There was also a lower percentage of females in the high-income group. Further, generic drugs were more commonly dispensed in the general-income group for all five drugs.

**Table 1:** Summary statistics on patients for Top 5 drugs in terms of number of prescriptions

	rebamipide 100 mg		amlodipine 5 mg		lansoprazole OD 15 mg		sennoside 12 mg		etizolam 0.5 mg	
	Copayment rate		Copayment rate		Copayment rate		Copayment rate		Copayment rate	
	10%	30%	10%	30%	10%	30%	10%	30%	10%	30%
Number of prescriptions	1,018,112	135,815	922,264	127,108	851,256	117,551	787,883	88,352	714,066	95,195
Generic dispensed	46.4%	44.1%^e^	50.8%	47.7%^e^	41.7%	38.3%^e^	49.0%	39.0%^e^	26.5%	23.1%^e^
Age, year										
Mean	82.9	82.3	83.2	82.4	84.5	83.7	85.3	84.8	83.3	82.5
Std. Dev.	5.3	5.1	5.5	5.2	5.9	5.6	6.0	5.7	5.3	5.1
Female	72.2%	56.5%	66.5%	48.1%	69.3%	51.3%	71.2%	51.0%	77.3%	61.8%
Amount^a^, tablets/capsules										
Mean	51.6	52.0	31.2	34.7	28.9	31.9	43.8	48.3	38.2	41.5
Std. Dev.	41.3	44.3	19.7	22.3	19.3	21.4	40.6	41.4	31.5	34.4
Medical spending^b, c^, JPY/month										
Mean	27,358	28,883	20,837	21,279	27,507	31,314	25,014	31,681	24,379	25,244
Std. Dev.	24,623	27,106	21,421	23,105	26,470	30,274	25,793	29,529	23,459	25,662
Spending on other drugs^b, d^, JPY/month										
Mean	25,534	25,637	21,258	21,745	27,723	29,779	23,400	26,886	23,949	24,410
Std. Dev.	23,669	26,258	19,538	21,628	25,276	28,081	25,458	28,794	22,327	23,813

Notes: Std. Dev. stands for standard deviation, OD stands for orally disintegrating tablet. We present summary statistics for top 5 prescribed drugs in number of prescriptions in our data. Statistics are calculated at the prescription level. The observation period is from October 2013 to September 2014. ^a^Amount is the total number of tablets/capsules within each prescription. ^b^“Medical spending” and “Spending on other drugs” are represented in Japanese yen (JPY) per month. 100 JPY is approximately 1 USD. Each spending is calculated as the sum of spending by the patient and the insurer. ^c^We exclude spending on drugs to calculate “Medical spending”. ^d^We exclude the drug to be analyzed in calculation of “Spending on other drugs”. ^e^The proportion of generic drugs dispensed was statistically smaller in the 30% copayment group (high-income group) than in the 10% copayment group (general-income group) (*p* < 0.01 for each of the 5 drugs)

### Results from analytical model 1

Hypothesis 1 was partly suggested because the odds ratios for choosing generic drugs of the high-income group to the general-income group were significantly less than 1 (i.e., ${\hat{\beta}}_k<0$) for the top-five prescribed drugs (Table 2). The fact that estimated odds ratio being smaller than 1 is not sufficient to completely support our Hypothesis 1, since the odds ratios subsume the effect of different out-of-pocket payment between income groups. The estimated odds ratios for five-top prescribed drugs ranged between 0.93 and 0.98 (four estimates with *p* < 0.001 and one estimate with *p* = 0.038). The estimated odds ratio (0.96) for rebamipide (in far left column in Table 2) indicated that patients with 30% copayment rate were 4% less likely to use a generic drug (or 4% more likely to use brand-name drug), compared to those with 10% copayment rate, after adjusting sex, age, prescribed amount, area of patient’s residence, monthly medical expenditure (excluding spending on drugs), and monthly spending on drugs besides rebamipide. In addition, females were observed to choose brand-name drugs more frequently.

**Table 2:** Estimation results for Top 5 drugs in terms of number of prescriptions

Dependent variable: Generic drug dispensed, Model: Logistic regression										
	rebamipide 100 mg		amlodipine 5 mg		lansoprazole OD 15 mg		sennoside 12 mg		etizolam 0.5 mg	
	OR	95% CI	OR	95% CI	OR	95% CI	OR	95% CI	OR	95% CI
Copayment rate										
10%	ref		ref		ref		ref		ref	
30%	0.96^a^	0.94–0.98	0.98^a^	0.97–1.00	0.96^a^	0.94–0.98	0.93^a^	0.91–0.95	0.94^a^	0.92–0.96
Sex										
Male	ref		ref		ref		ref		ref	
Female	0.85^a^	0.84–0.87	0.80^a^	0.78–0.82	0.85^a^	0.83–0.87	0.98	0.96–1.00	0.80^a^	0.77–0.82
Age, year										
75–79	ref		ref		ref		ref		ref	
80–84	0.89^a^	0.88–0.91	0.86^a^	0.83–0.88	0.93^a^	0.90–0.96	0.99	0.96–1.02	0.93^a^	0.90–0.96
85–89	0.81^a^	0.79–0.83	0.82^a^	0.80–0.85	0.96^a^	0.93–0.99	1.06^a^	1.03–1.10	0.90^a^	0.86–0.93
90-	0.82^a^	0.79–0.84	0.85^a^	0.82–0.89	1.07^a^	1.03–1.11	1.20^a^	1.16–1.24	1.01	0.96–1.06

Notes: OR is the odds ratio estimate, and 95% CI is the associated 95% confidence interval. “ref” indicates the reference group. OD stands for orally disintegrating tablet. This table shows the results from Eq. (1) described in the main text: logistic regression of a binary variable for generic drug dispensed on a 30% copayment rate dummy adjusting for individual characteristics conducted separately for each drug. Adjusted characteristics include sex, age, prescribed amount, area of patient’s residence, monthly medical expenditure (excluding spending on drugs), and monthly spending on drugs besides the analyzed drug. Each spending is calculated as the sum of spending by the patient and the insurer. We show results for the top 5 prescribed drugs in terms of number of prescriptions in our data. ^a^ indicates significance at the 5% level

**Fig. 1:**
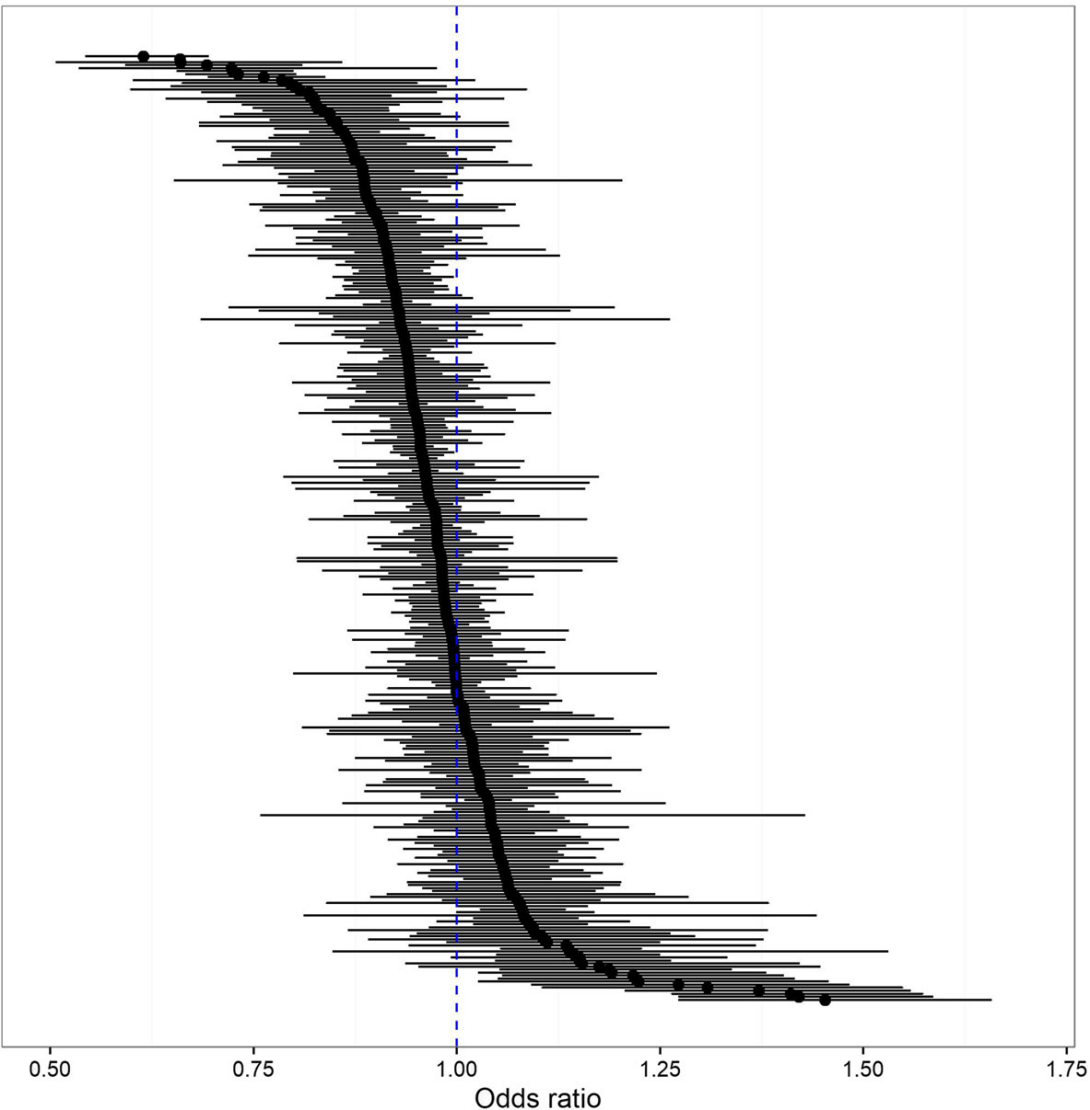
Odds ratios of choosing generic drugs between high- and general-income groups for 311 drugs. Notes: This figure shows odds ratios estimated from the logistic regression Eq. (1) described in the main text for the whole sample including 311 drugs: logistic regression of a binary variable for generic drug dispensed on a 30% co-payment rate (high-income group) dummy adjusting for individual characteristics conducted separately for each drug. Adjusted characteristics include sex, age, prescribed amount, area of patient’s residence, monthly medical expenditure (excluding spending on drugs), and monthly spending on drugs besides the analyzed drug. Each spending is calculated as the sum of spending by the patient and the insurer. Estimated odds ratios for each drug are shown in filled circles. 95% confidence intervals for each drug are shown in horizontal lines. Dotted line shows where odds ratio = 1 holds

Hypothesis 1 was again partly suggested because we found that 79 drugs had odds ratios significantly smaller than 1, while only 21 out of 311 drugs had odds ratios significantly larger than 1 (Fig. 1). Among the 311 drugs, the median of the estimated odds ratios was 0.97, and the interquartile range was 0.92–1.02.

### Results from analytical model 2

**Fig. 2:**
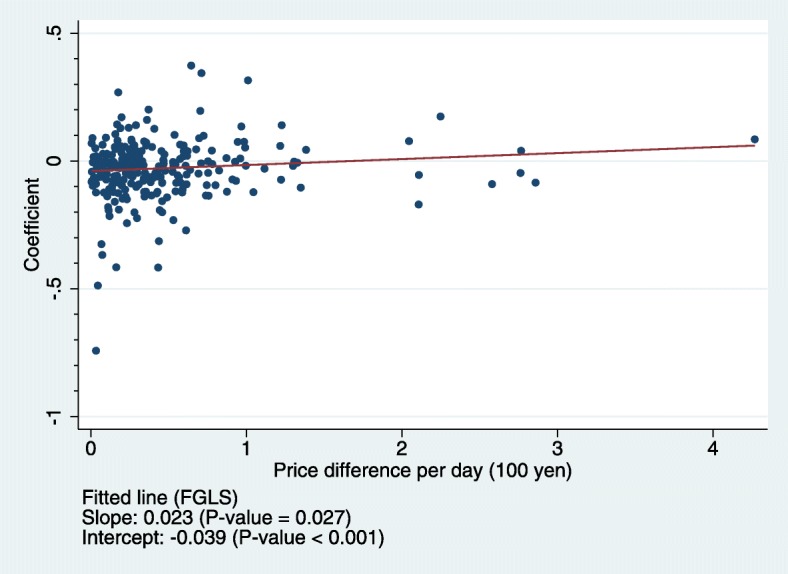
Association of brand-name/generic price difference with disparity in access to brand-name drugs for chronic conditions. Notes: This figure shows the association of price difference per day between brand-name and generic drugs with disparity in access to brand-name drugs for chronic conditions. Estimated coefficients of high-income group dummy for each drug from Eq. (1) address disparity in access to brand-name drugs (a coefficient of zero means no disparity). Each filled circle represents the 252 chronic drugs. The fitted regression line is obtained from FGLS regression of Eq. (2), which corresponds to column 6 in Table 3

Hypothesis 1 was supported since the estimated constant term in Eq. (2) was significantly negative, both in OLS and FGLS regressions (*p* < 0.001, Columns 1 and 2 of Table 3). The estimated constant term in Eq. (2) (− 0.034 for FGLS) indicated that the coefficient *β*_*k*_ would be − 0.034, suggesting patients with 30% copayment rate were 3.4% less likely to choose a generic drug compared to those with 10% copayment rate, if the price difference between brand-name and generic drugs was zero, equivalent to the case where brand-generic payment difference between income groups was zero. Furthermore, Hypothesis 2 was supported for chronic drugs, since the estimated coefficient of price difference in Eq. (2) was positive, both in OLS and FGLS regressions (*p* = 0.016 and *p* = 0.027, Columns 5 and 6 of Table 3). The estimated coefficient of price difference in Eq. (2) (0.023 for FGLS) indicated that an additional brand-generic price difference of 100 JPY/day, equivalent to additional out-of-pocket payment difference between income groups of 20 JPY/day, will decrease the difference in likelihood of choosing generic drugs between income groups by 2.3% (when patients with 30% copayment rate are less likely to choose generic drugs). Figure 2 provides a graphical illustration of the association of “price difference between brand-name and generic drugs” with “disparity in access to brand-name drugs for chronic drugs”. The fitted line of Eq. (2) shown in Fig. 2 implied that state of no disparity (*β*_*k*_ = 0) can be achieved under a feasible brand-generic price difference (100*(0.039/0.023) = 170 JPY/day), which is equivalent to feasible difference in additional out-of-pocket payment for a brand-name drug between income groups (100*(0.039/0.023)*(0.3–0.1) = 34 JPY/day). On the other hand, for acute drugs, the estimated coefficient of price difference in Eq. (2) was statistically insignificant, supporting Hypothesis 3 (*p* = 0.812 and *p* = 0.821, Columns 3 and 4 of Table 3). This insignificance indicated that brand-generic price difference, equivalent to out-of-pocket payment difference between income groups, had no significant effect on the difference in the likelihood of choosing generic drugs between income groups. In addition, our robustness-check analysis, adding further detailed drug category dummies as explanatory variables, also supported Hypothesis 1. This is because when we added such explanatory variables, the estimated constant term was significantly negative (*p* = 0.017 and *p* = 0.022) and the coefficients of drug category dummies were also negative, even though not all were statistically significant (Columns 7 and 8 of Table 3). The estimated constant term in Eq. (2) (− 0.023 for FGLS) and the estimated coefficients of drug category dummies (e.g., − 0.057 for nervous system drug category dummy in FGLS) indicated that the coefficient *β*_*k*_ would be − 0.080 (=(− 0.023) + (− 0.057)) for drugs categorized in the nervous system drug category, suggesting patients with 30% copayment rate were 8% less likely to choose a generic drug compared to those with 10% copayment rate, if the brand-generic price difference was zero.

**Table 3:** Results from regressing estimated 30% co-payment rate dummy coefficients from Eq.(1) on price difference

Dependent Variable: Estimated Coefficients $\hat{\upbeta_{\mathrm{k}}}$								
	Whole Sample		Acute Drugs		Chronic Drugs			
	(1)	(2)	(3)	(4)	(5)	(6)	(7)	(8)
	OLS	FGLS	OLS	FGLS	OLS	FGLS	OLS	FGLS
Price Difference (per 100JPY)	0.014^a^ (0.0072)	0.010^a^ (0.0062)	−0.0020 (0.0082)	−0.0018 (0.0079)	0.029^b^ (0.012)	0.023^b^ (0.010)	0.031^b^ (0.013)	0.022^b^ (0.011)
Constant	−0.039^b^ (0.0077)	−0.034^b^ (0.0060)	− 0.030^b^ (0.013)	−0.030^b^ (0.012)	− 0.047^b^ (0.0098)	−0.039^b^ (0.0075)	− 0.031^b^ (0.013)	−0.023^b^ (0.010)
ATC Categories								
Cardiovascular System	–	–	–	–	–	–	ref	ref
Alimentary Tract and Metabolism	–	–	–	–	–	–	−0.0059 (0.015)	−0.014 (0.012)
Nervous System	–	–	–	–	–	–	−0.059^b^ (0.023)	−0.057^b^ (0.020)
Others	–	–	–	–	–	–	−0.023 (0.022)	−0.014 (0.017)
Observations	311	311	59	59	252	252	252	252

Notes: “ref” indicates the reference group. We report the results from OLS and FGLS estimation of Eq. (2), which we regress the estimated coefficients of being in the high-income group dummy, i.e., having 30% co-payment rate, for each drug from Eq. (1) on price difference between brand-name and generic version of each drug. Columns (1) and (2) report the results from the whole sample which includes 311 drugs. Columns (3) and (4) report the results from the sample which includes 59 acute drugs. Columns (5) and (6) report the results from the sample which includes 252 chronic drugs. Columns (7) and (8) report the results from the sample which includes 252 chronic drugs, including the ATC category dummies as explanatory variables. “Cardiovascular System” category includes 96 drugs, “Alimentary Tract and Metabolism” category includes 56 drugs, and “Nervous System” category includes 43 drugs. The remaining 57 drugs are classified as “Others”. ^a^ indicates significance at the 10% level. ^b^ indicates significance at the 5% level

## Discussion

We found high-income group’s higher likelihood to choose brand-name drugs than the general-income group without co-payment rate differentiation. However, income-based co-payment rates appeared to reduce disparity in access to brand-name drugs across income groups, in addition to reducing total medical expenditure among high-income group who shifted from brand-name drugs to generic ones due to larger drug price differences.

Our results suggest that high-income population tend to prefer brand-name to generic drugs more than general-income population if co-payment rate is equal across income groups, supporting our Hypothesis 1. A study based on Danish data showed that access to brand-name drugs differs by income status, while out-of-pocket price of the drugs do not differ by their income status in Denmark. Our result follows the result from this previous study [6]. Additionally, reference-based pricing, which is a price scheme that the insurance covers cost up to a reference price, has been introduced in Denmark. In contrast to reference-based pricing, we showed that access to brand-name drugs also differs by income status in an environment where both the prices for brand-name and generic drugs are partially covered by health insurance.

The results also suggest that Japan’s policy of imposing higher co-payment rates for high-income elderlies than general-income ones improved disparity in terms of access to brand-name drugs relative to generic drugs between these two income groups, supporting our Hypothesis 2. Particularly, this policy effect was stronger when brand-generic price difference is larger. Our results suggest that imposing an additional out-of-pocket payment of 1000 JPY/month (approximately 10 USD/month) for brand-name drugs on high-income enrollees relative to general-income ones would result in a state of no gap in access to brand-name drugs between income groups.

Furthermore, although the policy efficacy was shown for drugs treating chronic conditions, the policy appeared to be ineffective for drugs treating acute conditions, supporting our Hypothesis 3. This result may be due to the fact that drugs for chronic conditions are repeatedly prescribed, which would provide patients with a larger number of opportunities to switch between brand-name and generic drugs, compared to drugs for acute conditions.

In our analysis for assessing the association between drug choice and co-payment rates, we controlled for individual characteristics. This allowed us to obtain precise estimates for disparity in terms of access to brand-name drugs between two income groups (*β*_*k*_ in Eq. (1)), strengthening the internal validity of our study. In addition, the positive estimate for the effect of Japan’s policy on improving disparity (*δ* in Eq. (2)) was robust to adding drug-category fixed effects as control variables, also indicating the high internal validity of the study. Furthermore, the magnitude of the difference in additional out-of-pocket payment (1000 JPY/month approximately equal to 10 USD/month) for brand-name drugs between income groups that would lead to a state of no disparity appears reasonable. This is because this magnitude is well within the range of the observed payments, i.e. 3–5000 JPY/month (approximately 0.03–50 USD/month).

Also, we expect our results to have high external validity. In our analysis, over 300 commonly prescribed drugs were analyzed. The targets of the analyzed drugs covered most of the major health conditions: chronic disease such as hypertension, dyslipidemia, and diabetes; acute conditions such as bacterial infections and acute pain. Thus, our results are expected to be valid for drugs not included in our analysis. Moreover, elderlies in Japan are mandatorily covered by one health insurance system. Therefore, there were no self-selection bias issues at the margin of obtaining insurance coverage (i.e., individuals cannot choose whether to be insured or not), strengthening external validity of our study.

Our analysis has several limitations. First, our data (between October 2013 and September 2014) did not allow us to exploit within-individual variation in co-payment rates by analyzing (a) individuals who switched between two income groups (i.e. two co-payment rates of 10 and 30%) or (b) individuals who experienced before/after the first implementation of the income-based co-payment (rate) schemes in Japan (April 2008). Disparity estimates (*β*_*k*_ in Eq. (1)) could be potentially biased upward compared to true estimates if enrollees with stronger preference for brand-name drugs had intentionally reduced their income to be eligible for the general-income group. Without the additional data addressed above, we cannot verify whether such income manipulation exists. However, our Hypothesis 1 would be supported stronger without such potential upward bias.

Second, variables such as the numerical income and education levels were not included due to the limitations of the available data. Compared to the true estimates for individual-level effect of imposing higher co-payment rate on the use of generic drugs, our disparity estimates (*β*_*k*_ in Eq. (1)) would be biased downward. Still, our qualitative conclusion regarding hypothesis 1 holds. Moreover, our conclusion regarding Hypothesis 2 (that is more important than Hypothesis 1 for our primary research goal) is not affected by such biases.

Third, in reality, patients may choose different drugs with “similar effects,” instead of switching between brand-name and generics with “same effects” examined in our analysis [17]. If such substitution across drugs with “similar effects” were prevalent, the disparity estimates (*β*_*k*_ in Eq. (1)) and the estimate for the effect of Japan’s policy on improving disparity (*δ* in Eq. (2)) could be biased. These biases’ directions are ambiguous, since they depend on both how enrollee’s income is distributed differs by drugs and how brand-name preference and income are correlated. However, although such bias may exist, the overall negative estimates for disparity suggest positive correlation between income and brand-name preference, which leads to a greater disparity without co-payment differentiation. In addition, the fact that some disparity estimates (21 out of 311) were significantly positive (*p* < 0.05) suggest that the effect of Japan’s policy is desirable in terms of reducing disparity, given high-income group has a stronger brand-name preference.

Fourth, our results are based on claims data from an area that includes a metropolitan area and a higher income population than the national average. This could lead to biased estimates of disparity (*β*_*k*_ in Eq. (1)) and biased estimate for the effect of Japan’s policy (*δ* in Eq. (2)). Again, these biases’ directions are ambiguous. However, although the magnitude of our disparity estimates may be biased, our results still suggest that high-income group has a stronger brand-name preference among any population if brand-name preference is monotonously increasing with income elsewhere in Japan. Also, the policy of copayment differentiation would still improve disparity among any population provided that the high-income group’s demand is price-elastic.

There is another point that is worth noting. The generic substitution rate has been increasing in Japan. This rate increased from 47% in 2013 to 73% in 2018 [11]. On the other hand, to our best knowledge, there is no study which reported how the high- and general-income population differed in terms of the change in this substitution rate during the same period. Thus, regarding our estimates on the policy impact based on the data from 2013 to 14, the magnitude of these estimates might not be applicable to more recent data in 2018. However, the direction of our estimates is expected to be applicable for more recent data as well. That is, as long as high-income population tend to prefer brand-name to generic drugs more than general-income population without copayment differentiation, the policy should still be improving disparity.

Schneeweiss and associates examined the effects of reference-based pricing on the choice of drugs within the same drug class. Low-income patients were more likely to switch from cost-shared drugs to drugs without cost within the same drug class than high-income patients [18]. We are not able to see the effects of the Japan’s policy on switching drugs due to our data limitation. However, our conclusion that the effect of Japan’s policy is desirable in terms of reducing disparity would be unchanged even if patients may choose different drugs with “similar effects” as discussed above.

Additionally, Caetano and associates examined worsened disparities in access to medicines, i.e., treatment initiation and treatment discontinuity, due to the implementation of income-based drug coverage [19]. Specifically, the introduction of income-based drug coverage had no significant impact on initiation and discontinuity of hypertension/dyslipidemia treatment across income groups. However, we are not able to see the effects of the Japan’s policy on treatment initiation and discontinuity due to our data limitation. Thus, we should interpret our results as the policy’s effect on enrollees who continued treatment.

Our study has important policy implications. First, if co-payment rate is equal among income groups, disparity in terms of access to brand-name drugs is inevitable. This disparity may stem partly from income effect and partly from difference in perceptions of generic drugs’ efficacy and safety between income groups. Since micro-level estimates for income elasticity tend to be small and even zero for some insured populations [20–22], we expect the size of income effect would be small in our analysis. Thus, providing information on generic drugs’ efficacy and safety to modify such perceptions could be effective to improve disparity provided income effect is negligible among the insured population. Second, imposing higher out-of-pocket payments (may be in the form of co-payment or co-payment rates) for brand-name drugs on the high-income group can improve disparity in access to brand-name drugs, particularly for drugs treating chronic conditions. If the policymaker is concerned about disparity in access to brand-name drugs, income-based co-payment (rate) schemes could be one solution. We expect such policy to be applicable and effective outside Japan.

## Conclusion

High-income group has a higher likelihood to choose brand-name drugs than the general-income group without co-payment rate differentiation. Japan’s policy that differentiates co-payment rate based on income reduced disparity in access to brand-name drugs across income groups. Our estimates suggest the state of no disparity would be achieved if high-income enrollees pay 10 USD/month additionally for brand-name drugs.

## Supplementary information

**Additional file 1.** Can income-based co-payment rates improve disparity? The case of the choice between brand-name and generic drugs.

## Data Availability

The datasets analyzed during the current study are not publicly available due to the contract with the Tokyo Extended Association of Medical Care System for the Latter-Stage Elderly People.

## Abbreviations

FGLS: Feasible generalized least squares
GEE: Generalized estimating equation
JPY: Japanese Yen
OECD: Organisation for Economic Co-operation and Development
OLS: Ordinary least squares
USD: US Dollar
