# Supplementary material for: Can income-based co-payment rates improve disparity? The case of the choice between brand-name and generic drugs
Source: BMC Health Serv Res. 2019 Nov 1;19:780. doi: 10.1186/s12913-019-4598-8 (PMC6824135; doi:10.1186/s12913-019-4598-8)
Supplement: Supplementary file 1 — Additional file 1. Can income-based co-payment rates improve disparity? The case of the choice between brand-name and generic drugs. [file 12913_2019_4598_MOESM1_ESM.pdf]

**Appendix for**

“Can income-based co-payment rates improve disparity? The case of the choice between brand-name and generic drugs”

**Appendix. Conceptual Model for Drug Choice and Calibration Studies for Utility Difference between Income Groups by Specifying the Utility Function and Income**

We model patient $i$'s utility $V_{ik}$ from brand-name and generic drug $k$ as follows:

$$V_{ik} = \begin{cases} U_i\left(Income_i - \alpha_i p_k^b\right) + \delta_{ik}^b, & \text{(brand} - \text{name chosen)} \\ U_i\left(Income_i - \alpha_i p_k^g\right) + \delta_{ik}^g, & \text{(generic chosen)} \end{cases}$$

where $\delta_{ik}^b, \delta_{ik}^g$ denote patient $i$'s utilities from brand-name and generic versions, $\alpha_i$ is patient $i$'s co-payment rate, and $p_k^b, p_k^g$ are the prices of brand-name and generic version of drug $k$. $Income_i$ is the income of patient $i$. $U_i(\cdot)$ is the patient's utility function for a monetary outcome. Since we focus on patients' choice after receiving a prescription, we assume there is no outside option besides choosing a brand-name or generic version of the drug. If $U_i\left(Income_i - \alpha_i p_k^b\right) + \delta_{ik}^b > U_i\left(Income_i - \alpha_i p_k^g\right) + \delta_{ik}^g$ holds, the patient will choose the brand-name drug, and vice versa. Our interest is the difference between $\delta_{ik}^b$ and $\delta_{ik}^g$ by a patient's income status. Furthermore, we aim to reveal the varying effects of price differences on patients' choice on the basis of income status.

We consider a simple setting. Assume there are two types of patients with high- and general-income ($i = h, g$, respectively), and $U_h = U_g$ $(= U)$ holds, that is, the utility function is common

across enrollees, irrespective of income. The generic version of drug $k$ is more likely to be chosen by the high-income group than the general counterpart if the following relationship holds:

$$P\left(U\left(Income_h - \alpha_h p_k^g\right) - U\left(Income_h - \alpha_h p_k^b\right) > \delta_{hk}^b - \delta_{hk}^g\right)$$

$$> P\left(U\left(Income_g - \alpha_g p_k^g\right) - U\left(Income_g - \alpha_g p_k^b\right) > \delta_{gk}^b - \delta_{gk}^g\right),$$

where $P(A)$ indicates the probability of event $A$. Figure A1 illustrates our model.

For the sake of illustration, we compare the difference in $U\left(Income_i - \alpha_i p_k^g\right) - U\left(Income_i - \alpha_i p_k^b\right)$ between $i = h$ and $i = g$ under certain utility function specifications. If we define $I_{ik} = Income_i - \alpha_i p_k^g$ for $i = h, g$ and $p_k = p_k^b - p_k^g$, we can rewrite this term as $U(I_{ik}) - U(I_{ik} - \alpha_i p_k)$. We describe the results of certain calibration exercises for the size of $U\left(Income_i - \alpha_i p_k^g\right) - U\left(Income_i - \alpha_i p_k^b\right)$.

Table A1 reports the results from a calibration exercise. We assume a Constant Relative Risk Aversion (CRRA) utility $U(x) = (x^{1-\theta} - 1)/(1 - \theta)$. In our setting, $\alpha_h = 0.3$ and $\alpha_g = 0.1$ holds. We report three panels for three different values of CRRA parameter $\theta$. Columns 1 and 2 report the value of $U(I_{ik}) - U(I_{ik} - \alpha_i p_k)$ for $i = h$, columns 3 and 4 for $i = g$. We report this amount for several values of $I_{ik}$ and $p_k$. As we can see from this result, whether $U(I_{ik}) - U(I_{ik} - \alpha_i p_k)$ is larger for $i = h$ or $i = g$ depends on the form of the utility function and income related term $I_{ik}$. When $\theta = 1$, $U(I_{ik}) - U(I_{ik} - \alpha_i p_k)$ tend to be larger in the higher

income group in our setting of income. In contrast, when $\theta = 2, 3$, the results suggest that this comparison in size heavily depends on income of each group.

Our calibration results suggest that whether the size of $U(I_{ik}) - U(I_{ik} - \alpha_i p_k)$ is larger for high- or general-income enrollees remains controversial and the size can be substantial. In other words, there is no clear implication for how the difference in probability of choosing generic drugs between high- and general-income groups responds to price changes and the difference may be sizeable such that equal probability cannot be achieved within a realistic co-payment rate setting. In addition, it is important to know the distributions of $\delta_{ik}^b, \delta_{ik}^g$ to elucidate the differences in preferences for brand-name drugs by income status. By empirically assessing these aspects, we can determine whether differentiating co-payment rates can contribute to the equal probability of high- and general-income groups choosing generic drugs.

**Appendix. Details of the Patients' Choice Model and Empirical Strategy**

We describe the relations between our empirical methods and an underlying patients' choice model. We denote the patient $i$'s utility from choosing each version of the drug and introduce time dimension *t*:

$$V_{ikt} = \begin{cases} U(I_{ik} - \alpha_i p_k) + \delta^b_{ikt}, & \text{(brand} - \text{name chosen)} \\ U(I_{ik}) + \delta^g_{ikt}, & \text{(generic chosen)} \end{cases}$$

where we assume that the patient's utility function $U$ is time-invariant.

We decompose $\delta^j_{ikt}$ $(j = b, g)$ as follows:

$$\delta^j_{ikt} = \mu^j_k + D_{it}\pi^j_k + X_{ikt}\gamma^j_k + \varepsilon^j_{ikt},$$

where $b$ denotes brand-name drug and $g$ indicates generic drugs; $\mu^j_k$ is the drug-version-specific time-invariant preference toward version *j*; $D_{it}$ is a dummy variable that equals 1 if patient $i$'s co-payment rate is 30%, which also indicates a relatively high income; $X_{ikt}$ is a vector for other individual characteristics; and $\varepsilon^j_{ikt}$ is an error term for an unobservable taste shock. Coefficient $\pi^j_k$ is the change in preference for version *j* caused by being in the high-income group instead of the general-income group. We allow each patient's preference to differ by their income level and other observable characteristics. Let $\delta_{ikt} = \delta^g_{ikt} - \delta^b_{ikt}, \mu_k = \mu^g_k - \mu^b_k,$ $\gamma_k = \gamma^g_k - \gamma^b_k,$ $\varepsilon_{ikt} = \varepsilon^g_{ikt} - \varepsilon^b_{ikt},$ $\pi_k = \pi^g_k - \pi^b_k$. Note that $\pi_k$ can be interpreted as the difference in preference

for the generic version relative to the brand-name version between the high- and general-income groups. Assuming $U(I_{ikt}) - \mathrm{U}(I_{ikt} - \alpha_i p_k) = \alpha_i p_k U'(I_{ikt})$ by linear approximation, we rewrite the condition for the generic version to be chosen as:

$$\alpha_i p_k U'(I_{ik}) + \mu_k + D_{it}\pi_k + X_{ikt}\gamma_k + \varepsilon_{ikt} > 0.$$

Here, recall that we assume two types of patients with high or general income adjusted to the price of generic drugs $I_{hk}, I_{gk}$. Finally, the condition can be written as

$$D_{it}\{p_k(\alpha_h U'(I_{hk}) - \alpha_g U'(I_{gk})) + \pi_k\} + \alpha_g p_k U'(I_{gk}) + \mu_k + X_{ikt}\gamma_k + \varepsilon_{ikt} > 0.$$

Assuming that $\varepsilon_{ikt}^{g}$ and $\varepsilon_{ikt}^{b}$ are exogenous error terms with type-I extreme value distribution, this condition motivates us to conduct a logistic regression for each drug as follows:

$$\boldsymbol{Logit}(\boldsymbol{P}(\boldsymbol{y}_{ikt} = \mathbf{1})) = \widetilde{\boldsymbol{\mu}_k} + \boldsymbol{\beta}_k \boldsymbol{D}_{it} + \boldsymbol{X}_{ikt}\boldsymbol{\gamma}_k, \qquad \{1\}$$

where dependent variable $y_{ikt}$ is a dummy variable that equals 1 if a generic version of drug $k$ is dispensed for patient $i$ at prescription timing $t$. We include a set of covariates for $X_{ikt}$ such as age, sex, amount of drug prescribed at $t$, area of patient's residence, total medical expenditure in a month including prescription timing $t$ but excluding spending on drugs, and total spending on drugs besides drug $k$ in a month including prescription timing $t$. $\widetilde{\mu_k}$ is an intercept (we rewrite $\alpha_g p_k U'(I_{gk}) + \mu_k$ as $\widetilde{\mu_k}$). Since individuals tend to receive a prescription for the same drug repeatedly, we employ a generalized estimating equation (GEE) approach to account for the

clustering responses among the same individuals. To model the within-individual correlation, we choose a working correlation matrix to have an exchangeable structure.

Our central estimate of interest is $\beta_k$. If this coefficient is smaller than 0 (i.e., the odds ratio is less than 1), being in the high-income group reduces the probability of choosing generic drugs, despite the higher out-of-pocket prices. From the above argument, $\beta_k$ comprises two components:

$$\boldsymbol{\beta_k = p_k(\alpha_h U'(I_{hk}) - \alpha_g U'(I_{gk})) + \pi_k}, \qquad \{2\}$$

where $p_k(\alpha_h U'(I_{hk}) - \alpha_g U'(I_{gk}))$ is the difference in utility loss from the high price of a brand-name version between high- and general-income groups and $\pi_k$ is difference in preference for a generic drug between high- and general-income groups. We focus on the role of these components in the choice of versions.

We analyze the relationship between estimated coefficients $\widehat{\beta_k}$ and price difference between the brand-name and generic drugs. We define price difference as the product of price difference per unit dose and average daily dose. To analyze the relationship, we regress the estimated coefficient $\widehat{\beta_k}$ on price difference. We introduce certain assumptions to interpret our results from the regressions.

**Assumption 1.** The difference in utility loss from the price difference per one yen between high- and general-income groups, $(\alpha_h U'(I_{hk}) - \alpha_g U'(I_{gk}))$, is the same across drugs $k$.

Although this assumption is untestable using our data, the drugs included in our analysis are commonly prescribed drugs. Therefore, we consider this assumption to be reasonable as the income level would not largely differ among such drugs.

**Assumption 2a.** The difference in preference for generic drugs between high- and general-income groups, $\pi_k$, is heterogeneous with mean $\pi$. In addition, $\pi_k$ is mean-independent of $p_k$, that is, $\mathrm{E}(\pi_k - \pi | p_k) = 0$ holds.

It is natural that the preference for generic drugs is heterogeneous among drugs. Although the conditional mean-zero assumption may be strong, we make this assumption for inference. To relax this assumption to a certain extent, we later allow mean $\pi$ to differ among the types of drugs. Under assumptions 1 and 2a, the price coefficient can be interpreted as $\alpha_h U'(I_{hk}) - \alpha_g U'(I_{gk})$ and the intercept as $\pi$.

Next, we regress $\widehat{\beta_k}$ on price difference and drug category dummies. To interpret the results from this regression, we relax Assumption 2a and instead, propose the following assumption.

**Assumption 2b.** The difference in preference for generic drugs between the high- and general-income groups, $\pi_k$, is heterogeneous with mean $\pi_c$ in each drug category, $c$. In addition, we assume mean independence between $\pi_k$ and $p_k$ within each drug category $c$, that is, $\mathrm{E}(\pi_k - \pi_c | p_k) = 0$ holds.

Instead of Assumption 2a, we allow the mean level of preference to differ by drug category. Again, under assumptions 1 and 2b, the price coefficient can be interpreted as $\alpha_h U'(I_{hk}) - \alpha_g U'(I_{gk})$ and the intercept as $\pi_c$ of the reference category $c$ in the regression.

**Appendix. FGLS Estimation**

We describe the FGLS estimation procedure which follows the previous literature on estimating regression models where the dependent variables are estimates. Our model of interest is:

$$\beta_k = \theta p_k + \pi + u_k$$

where we rearrange the terms $\theta = \alpha_h U'(I_h) - \alpha_l U'(I_l)$ and $\pi + u_k = \pi_k$. We introduced Assumption 1 to drop index k in the terms $I_{hk}$ and $I_{lk}$. From Assumption 2, $u_k$ is assumed to be mean-zero conditional on $p_k$ and let the variance be $\sigma^2$. Now, as $\beta_k$s are estimated in our model, let the estimates be $\widehat{\beta_k}$. Let

$$\widehat{\beta_k} = \beta_k + e_k.$$

In addition, assume that $Var(e_k) = \omega_k^2$. We assume that the estimate of the $\beta_k$ for each observation is independent of the others, in other words, we assume $Cov(e_k, e_l) = 0$ where $k \neq l$. By substituting this equation we obtain:

$$\widehat{\beta_k} = \theta p_k + \pi + u_k + e_k.$$

Let $v_k = u_k + e_k$. Also, let

$$G = \begin{bmatrix} \omega_1^2 & \cdots & 0 \\ \vdots & \ddots & \vdots \\ 0 & \cdots & \omega_K^2 \end{bmatrix}.$$

Following Lewis & Linzer (2005), we first calculate residuals $\widehat{v_k}$ from OLS regression. Then, we can obtain a consistent estimator for $\sigma^2$ as:

$$\widehat{\sigma^2} = \left\{ \sum_k \widehat{v_k}^2 - \sum_k \omega_k^2 + tr((X'X)^{-1}X'GX) \right\} \Big/ (K-2)$$

where

$$\mathrm{X} = \begin{bmatrix} 1 & p_1 \\ \vdots & \vdots \\ 1 & p_K \end{bmatrix}.$$

Assuming that we have consistent estimators for $\omega_k$ , which is the standard errors from the logistic regression, we may substitute the standard errors in the equation above without harming consistency of $\widehat{\sigma^2}$. Note that the variance-covariance matrix for $\mathrm{v} = (v_1, v_2, \dots, v_K)'$ is:

$$\mathrm{E(vv')} = \mathrm{V} = \begin{bmatrix} \sigma^2 + \omega_1^2 & \cdots & 0 \\ \vdots & \ddots & \vdots \\ 0 & \cdots & \sigma^2 + \omega_K^2 \end{bmatrix}.$$

By substituting estimates for $\sigma^2$ and $\omega_k$ , we can conduct FGLS estimation using this matrix as the weighting matrix.

**Figure A1.** Conceptual framework: utility loss from paying high prices for brand-name drugs by income status

Notes: The utility function is drawn in this figure. What we compare here is $U(I_i) - U(I_i - \alpha_i p)$ pointed out in the figure.

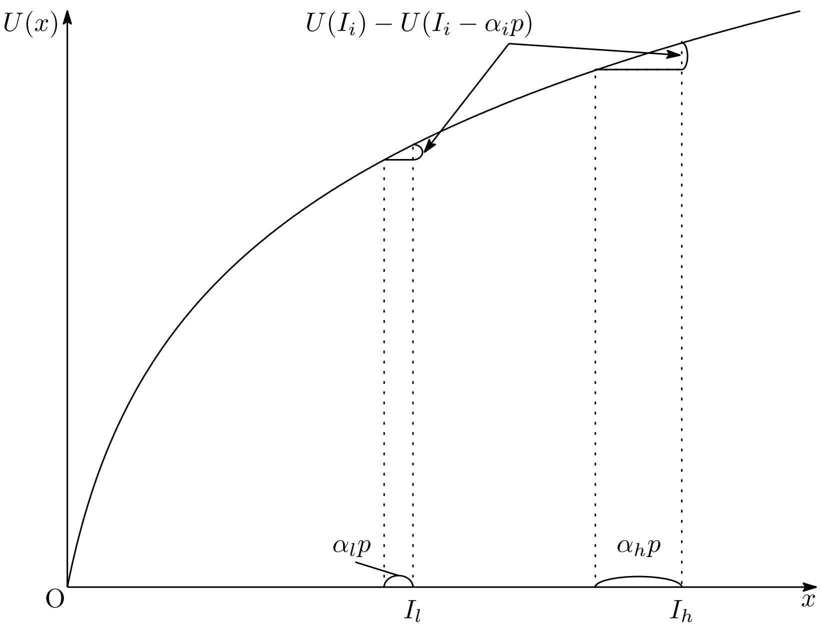
U(x)
U(I_i) − U(I_i − α_i p)
α_l p
α_h p
O
I_l
I_h
x

**Table A1**

Calibration results – Utility differences for different specifications of parameters and prices

| | High-income group | | General-income group | |
|---|---|---|---|---|
| Income | 5,000,000 | 4,000,000 | 3,000,000 | 2,000,000 |
| Price difference | CRRA parameter = 1 | | | |
| 1,000 | 6.000 | 7.500 | 3.333 | 5.000 |
| 3,000 | 18.002 | 22.503 | 10.001 | 15.001 |
| 10,000 | 60.018 | 75.028 | 33.339 | 50.013 |
| 30,000 | 180.162 | 225.254 | 100.050 | 150.113 |
| | CRRA parameter = 2 | | | |
| 1,000 | 1.200 | 1.875 | 1.111 | 2.500 |
| 3,000 | 3.601 | 5.626 | 3.334 | 7.501 |
| 10,000 | 12.007 | 18.764 | 11.115 | 25.013 |
| 30,000 | 36.065 | 56.377 | 33.367 | 75.113 |
| | CRRA parameter = 3 | | | |
| 1,000 | 2.400 | 4.688 | 3.704 | 12.501 |
| 3,000 | 7.202 | 14.067 | 11.113 | 37.508 |
| 10,000 | 24.022 | 46.928 | 37.056 | 125.094 |
| 30,000 | 72.195 | 141.101 | 111.278 | 375.845 |

Notes: This table presents the values of $U(I_{ik}) - U(I_{ik} - \alpha_i p_k)$ for each pairs of income $I_{ik}$, price difference $p_k$, and Constant Relative Risk Aversion (CRRA) parameters. $\alpha_i$ is set at 0.3 and 0.1 for the high- and general-income group.
